# Nosocomial Bloodstream Infection and Clinical Sepsis

**DOI:** 10.3201/eid1001.030407

**Published:** 2004-01

**Authors:** Stéphane Hugonnet, Hugo Sax, Philippe Eggimann, Jean-Claude Chevrolet, Didier Pittet

**Affiliations:** * University of Geneva Hospitals, Geneva, Switzerland

**Keywords:** Bloodstream infection, sepsis, nosocomial infection, surveillance, benchmarking

## Abstract

Primary bloodstream infection (BSI) is a leading, preventable infectious complication in critically ill patients and has a negative impact on patients’ outcome. Surveillance definitions for primary BSI distinguish those that are microbiologically documented from those that are not. The latter is known as clinical sepsis, but information on its epidemiologic importance is limited. We analyzed prospective on-site surveillance data of nosocomial infections in a medical intensive care unit. Of the 113 episodes of primary BSI, 33 (29%) were microbiologically documented. The overall BSI infection rate was 19.8 episodes per 1,000 central-line days (confidence interval [CI] 95%, 16.1 to 23.6); the rate fell to 5.8 (CI 3.8 to 7.8) when only microbiologically documented episodes were considered. Exposure to vascular devices was similar in patients with clinical sepsis and patients with microbiologically documented BSI. We conclude that laboratory-based surveillance alone will underestimate the incidence of primary BSI and thus jeopardize benchmarking.

Primary bloodstream infection (BSI) is a leading, infectious complication among critically ill patients (*1*). It represents about 15% of all nosocomial infections (*2*,*3*) and affects approximately 1% of all hospitalized patients (*4*), with an incidence rate of 5 per 1,000 central-line days (*5*). The impact on patient outcome is tremendous; BSI increases the mortality rate (*6*,*7*), prolongs patient stay in an intensive care unit (ICU) and in the hospital (*7*–*9*), and generates substantial extra costs (*7*,*8*). For these reasons, surveillance and prevention of BSI are high priorities, and several interventions have proven to be effective (*10*–*16*).

The Centers for Disease Control and Prevention (CDC) surveillance definitions of BSI delineate two distinct entities: infections that are microbiologically documented, and those that are not, called clinical sepsis (*17*). Although surveillance of the former can be laboratory based, detection of clinical sepsis requires prospective on-site surveillance. The surveillance strategy determines whether clinical sepsis will be detected, thus affecting the overall BSI incidence rate.

Because prospective on-site surveillance requires more resources than laboratory-based surveillance, the choice of the surveillance strategy should be based on knowledge of the importance of clinical sepsis. To our knowledge, clinical sepsis has never been investigated. This article describes the epidemiology of clinical sepsis in a medical ICU.

## Methods

### Setting

The study took place in the 18-bed medical ICU of a large teaching hospital in Geneva, Switzerland, from October 1995 to November 1997. The unit admits 1,400 patients per year; the mean length of stay is 4 days.

### Surveillance and Definitions

The surveillance strategy of nosocomial infection has been described previously (*12*). Briefly, one infection control nurse visited the ICU daily (5 of 7days), gathered information from medical and nursing records, microbiologic and x-ray reports, and interviews with nurses and physicians in charge. All patients staying >48 hours were included and followed up for 5 days after ICU discharge (*18*). Nosocomial infections were defined according to CDC criteria (*17*), except that asymptomatic bacteriuria was not considered an infection (*19*). Collected variables included all nosocomial infections, demographic characteristics, admission and discharge diagnoses, exposure to invasive devices and antibiotics, and ICU and hospital survival status.

Microbiologically documented BSI required one of the following: 1) recognized pathogen in the blood and pathogen not related to an infection at another site; or 2) fever, chills, or hypotension; and any of the following: a) a common skin contaminant is isolated from at least two blood cultures drawn on separate occasions, and the organism is not related to infection at another site; b) a common skin contaminant is isolated from blood culture in a patient with an intravascular device, and the physician institutes appropriate antimicrobial therapy; c) a positive antigen test on blood and the organism is not related to infection at another site (*17*).

Clinical sepsis was diagnosed when the patient had either fever, hypotension, or oliguria, and all of the following: 1) blood not cultured or no microorganism isolated; 2) no apparent infection at another site; and 3) physician institutes appropriate antimicrobial therapy for sepsis (*17*).

The surveillance strategy, definitions, and the discharge policy did not change over the study period. Patients were discharged from the ICU, according to specific guidelines designed for this unit, and compliance with these guidelines was checked daily by a senior staff member. An ongoing intervention aiming to reduce catheter-related infection was begun in March 1997. Reports on the intervention and its effect have been published previously (*12*).

### Statistical Analysis

All primary BSI were considered in the first part of the analysis. Episodes of BSI that were not associated with a central line were identified. Infection rates were expressed as the total number of episodes per 1,000 ICU patient days, or the number of episodes associated with a central line per 1,000 central-line days. Their corresponding 95% confidence intervals (CI) were computed, according to the normal approximation of the Poisson distribution.

The study population was then divided into three groups to describe the epidemiology of clinical sepsis. The first group included all patients who remained free of any ICU-acquired BSI; the second group comprised all patients whose first episode was a microbiologically documented BSI, and the third group included those whose first episode was clinical sepsis. Only the first episode of BSI was considered. We then performed a subgroup analysis comparing patients with and without BSI but with at least a 5-day stay in the ICU. This analysis was conducted to exclude patients who died or were discharged quickly after ICU admission to ensure that patients without BSI were sufficiently exposed to the risk of acquiring nosocomial BSI.

Exposure to invasive devices was estimated by the proportion of patients exposed to the device and the duration of the exposure. We separately investigated peripheral, arterial, and central vascular lines. Among patients with BSI, the duration of the exposure to the vascular line was censored at onset of the first episode of BSI.

Continuous variables were summarized by means or medians and compared with the Student t-test or a nonparametric test, when appropriate. Categorical variables were compared by using chi-square or the Fisher exact test. All tests were two-tailed, and p values <0.05 were considered statistically significant. All statistical analyses were conducted with Stata 7.0 (Stata Corporation, College Station, TX).

## Results

We surveyed the records of 1,068 patients who stayed in the ICU >48 hours, for a median length of stay of 5 days (range 2–134), totaling 7,840 ICU patient days. Median age was 62.9 (range 16.2–92.0), and male-to-female ratio 622/446. The main admission diagnoses were infectious (38.7%), cardiovascular (24.2%), and pulmonary (17.7%) conditions. We detected 554 ICU-acquired infections, yielding an infection rate of 71 episodes per 1,000 patient-days (95% CI 64.8 to 76.5). The leading sites were the lungs (pneumonia, 28.7%), bloodstream (20.4%), skin and soft tissue (15.3%), catheter exit site (13.5%), and urinary tract (11.2%). We detected nine episodes of secondary BSI, six secondary to a urinary tract infection, two to a lower respiratory tract infection, and one to a skin and soft tissue infection.

Of 113 episodes of BSI, 33 (29.2%) were microbiologically confirmed, and 80 (70.8%) were clinical sepsis. Four episodes (three of clinical sepsis and one of microbiologically confirmed BSI) were not associated with a central line. Blood cultures were drawn in most of the clinical sepsis episodes (66/80, 82.5%). Exposure to systemic antimicrobial drugs before blood culture was 39.4% (13/33) among patients with microbiologically documented BSI and 77.3% (51/66) among patients with clinical sepsis (p < 0.001). Among the 20 patients with microbiologically documented BSI who had not received antimicrobial drugs during the 48 hours before the blood culture, 6 were in a therapeutic window; antibiotherapy was suspended before drawing blood cultures to increase the culture’s sensitivity.

Among the 33 episodes of microbiologically confirmed BSI, 4 were polymicrobial. The most frequently isolated microorganisms were coagulase-negative staphylococci (n = 21). Other gram-positive cocci were Staphylococcus aureus (n = 1) and Enterococcus faecalis (n = 2). Gram-negative rods included Enterobacter aerogenes (n = 2), Serratia marcescens (n = 2), Escherichia coli (n = 1), Proteus mirabilis (n = 1), and Pseudomonas non-aeruginosa (n = 1). Other microorganisms found were Candida albicans (n = 1) and Propionibacterium acnes (n = 2).

Table 1 displays BSI infection rates per 1,000 patient days and central-line days. The overall rate of BSI was 19.8 per 1,000 central-line days (CI 95%, 16.1 to 23.6) and markedly differed when only microbiologically documented BSI were considered. These 113 BSIs occurred in 91 patients; 73 patients had a single episode, 14 had two, and 4 had three episodes. The first episode was microbiologically documented for 28 patients and diagnosed as clinical sepsis for 63.

**Table 1:** Primary bloodstream infection rates^a^

	N	Incidence rate/1,000 patient days (CI 95%)	N	Incidence rate/1,000 central-line days (CI 95%)
All primary bloodstream infections	113	14.4 (11.8 to 17.1)	109	19.8 (16.1 to 23.6)
Microbiologically documented	33	4.2 (2.8 to 5.6)	32	5.8 (3.8 to 7.8)
Clinical sepsis	80	10.2 (8.0 to 12.4)	77	14.0 (10.9 to 17.1)

^a^CI; confidence interval.

Selected characteristics of patients with and without BSI are displayed in Table 2. Patients without BSI tended to be older; the distribution of admission diagnosis was similar in both groups, but intoxication was more prevalent in patients without BSI, although the difference was not statistically significant. Illness appeared more severe in patients with BSI, as estimated by a higher number of discharge diagnoses, a longer ICU length of stay, and a higher mortality rate. After patients who stayed <5 days were excluded, 558 patients remained in this analysis. The picture remained the same. In particular, both groups were of similar age (p = 0.054); the proportion of patients admitted for intoxication was 1.9% in those without BSI and 2.3% in patients with BSI (p = 0.82).

**Table 2:** Selected characteristics of the study population^a^

	Patients without BSI n = 977	Patients with BSI n = 91	p value
Sex			0.28
Male (%)	562 (57.5)	60 (65.9)	
Female (%)	415 (42.5)	31 (34.1)	
Median age (range)	63.0 (16.2–92.0)	59.2 (18.7–86.8)	0.05
Admission diagnosis			
Infectious (%)	377 (38.6)	36 (39.6)	0.86
Cardiovascular (%)	241 (24.7)	17 (18.7)	0.2
Pulmonary (%)	171 (17.5)	18 (19.8)	0.59
Neurologic (%)	68 (7.0)	10 (11.0)	0.16
Intoxication (%)	50 (5.1)	2 (2.2)	0.22
Others (%)	70 (7.2)	8 (8.8)	0.57
No. of discharge diagnoses (range)	5 (1–30)	6 (1–19)	<0.001
ICU length of stay (range)	4 (2–134)	14 (3–67)	<0.001
ICU mortality rate	154 (15.8)	25 (27.5)	0.004

^a^BSI, bloodstream infection; ICU, intensive care unit.

The occurrence of pneumonia, urinary tract infection, and other infections was similar in patients with microbiologically documented BSI and clinical sepsis, but less frequent in patients without BSI. However, catheter exit-site infection was more frequent in patients with clinical sepsis (Figure).

**Figure:**
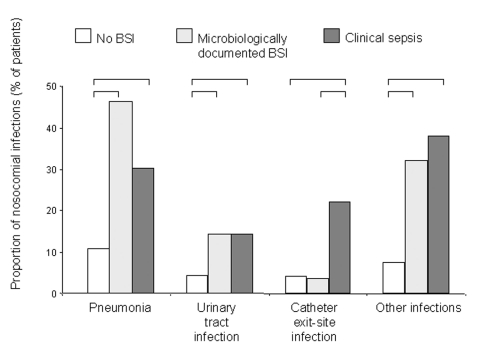
Frequency of nosocomial infections among patients with and without primary bloodstream infection. Columns represent the proportion of patients with each type of infection. Brackets indicate a significant (p < 0.05) difference between groups.

The results of exposure to invasive devices are shown in Table 3. Exposure to vascular lines was censored at the time of the first episode of BSI. Exposure to central lines and arterial lines was similar in patients with a microbiologically documented episode of BSI and in those with clinical sepsis but much lower in patients without BSI. Three episodes of primary BSI occurred in patients without a central line in place before onset of infection. Similarly, exposure to urinary catheter and mechanical ventilation was lower in patients without BSI. After patients who stayed <5 days in the ICU were excluded, exposure to central vascular lines remained more important in patients with BSI (96.6% of exposed patients vs. 76.4%, p < 0.001), and duration of the exposure was also longer in this group (median [range], 9 days [1-39], vs. 7 days [1-117], p = 0.002).

**Table 3:** Exposure to invasive devices among patients with and without primary bloodstream infection^a^

	No BSI n = 977	Microbiologically confirmed BSI n = 28	Clinical sepsis n = 63
Peripheral catheter			
Exposed patients (%)	858 (87.8)	24 (85.7)	58 (92.1)
Catheter-days [days, median (range)]	3 (1-30)	4 (1-10)^b^	5.5 (1-20)^c^
Central line			
Exposed patients (%)	627 (64.2)^d^	27 (96.4)	61 (96.8)
Catheter-days [days, median (range)]	4 (1-117)^d^	8 (2-39)	8 (1-33)
Arterial line			
Exposed patients (%)	791 (81.0)^d^	28 (100)	62 (98.4)
Catheter-days [days, median (range)]	3 (1-47)^d^	7 (2-23)	8 (1–21)
Mechanical ventilation			
Exposed patients (%)	380 (38.9)^d^	19 (67.9)	53 (84.1) ^e^
MV-days [days, median (range)]	3 (1–123)^d^	12 (2–61)	11 (1–35)
Urinary catheter			
Exposed patients (%)	665 (68.1)^d^	27 (96.4)	58 (92.1)
Catheter-days [days, median (range)]	3 (1–77)^d^	12 (1–63)	14 (1–45)

^a^BSI, bloodstream infection; MV, mechanical ventilation. ^b^p = 0.059 when compared to no BSI. ^c^p < 0.001 when compared to no BSI, and p = 0.053 when compared to microbiologically confirmed BSI. ^d^p < 0.005 when compared to microbiologically confirmed BSI and clinical sepsis. ^e^p = 0.097 when compared to microbiologically confirmed BSI.

Median ICU length of stay was longer among patients with microbiologically documented BSI (15.5 days; range 4–67) and clinical sepsis (14.0 days; range 3–48) than among patients with no BSI (4 days; range 2–134), (both p < 0.001). The hospital mortality rates among patients without BSI, with a microbiologically confirmed BSI, and with clinical sepsis were 22.7%, 32.1%, and 39.7%, respectively; the difference was statistically significant between the first and last group (p = 0.01).

## Discussion

This study shows the importance of primary BSI; the bloodstream was the second most frequent infection site, representing 20% of all infections. We also found that a minority of BSI were microbiologically documented and that ignoring clinical sepsis has a large impact on the BSI infection rate. To our knowledge, this is the first report that provides a detailed epidemiologic description of clinical sepsis.

Whether clinical sepsis represents a primary BSI or whether it is a systemic reaction accompanying an unrecognized infection at another site or a noninfectious systemic inflammatory response are valid concerns (*1*,*20*–*23*). The definition is not specific because it requires, among other criteria, only one of three clinical signs (fever, hypotension, or oliguria). Also, this condition mandates antimicrobial therapy prescribed by the physician for suspected sepsis. Thus, we decided to use unmodified definitions, elaborated by CDC and widely used because they are still considered the standard operational definitions for surveillance of nosocomial infections. An epidemiologic description of patients without BSI, with microbiologically documented BSI, and with clinical sepsis provides valuable information. First, approximately 90% of primary BSIs occur in patients with intravascular devices, especially central lines, and these represent the most powerful risk factors for BSI (*24*). In our study population, exposure to central and arterial lines was similar in both groups of patients with BSIs, but the frequency and duration of the exposure were of greater importance than they were in the group of patients without BSIs. The longer exposure to vascular devices does not reflect the impact of BSI because exposure was censored at time of BSI. Consequently, the most powerful risk factor for clinical sepsis is the same as that for microbiologically documented BSI.

Second, during the same study period we implemented an intervention targeted at vascular-access care to reduce the incidence of catheter-related BSIs (*12*). We observed a dramatic decrease in the incidence of all catheter-related infections: catheter exit-site infection dropped from 9.2 to 3.3 episodes per 1,000 ICU-patient days (64% reduction), and microbiologically documented BSI dropped from 3.1 to 1.2 episodes per 1,000 ICU-patient-days (61% reduction). A parallel sharp decrease occurred in the rate of clinical sepsis, which went from 8.2 to 2.6 episodes per 1,000 ICU-patient days (68% reduction). Rates of ventilator-associated pneumonia and urinary tract infection did not change over time. These two sets of results, same exposure and same response to a prevention program, strongly suggest that clinical sepsis is indeed primary BSI.

Blood cultures were performed in most (82.5%) cases of clinical sepsis and were negative. The absence of microorganisms can be explained in several ways. First, bacteremia is not constant, and sensitivity of the blood culture increases with the number of cultures drawn and the volume of the sample (*25*–*27*). Second, most of our patients (77%) with clinical sepsis were receiving broad-spectrum antimicrobial drugs for other conditions, thus decreasing the sensitivity of the test. This pattern of antimicrobial prescription is usual in critical care, as reported in large studies which showed that >60% of the patients were receiving antimicrobial drugs on the day of the study (*2*,*28*,*29*). In further studies to delineate the epidemiology and pathophysiology of clinical sepsis, the sensitivity of blood cultures should be maximized and should include genomic approaches to identify pathogens, especially if antimicrobial therapy has been initiated.

The question arises regarding whether to include clinical sepsis in surveillance of BSI, considering the amount of work generated by on-site prospective surveillance, compared to laboratory-based surveillance. In response, the following elements should be considered. Benchmarking is increasingly performed and is part of the quality improvement process. However, the sensitivity of the surveillance method to detect clinical sepsis will greatly impact the infection rate and make benchmarking difficult. Our overall BSI rate was high (19.8 episodes per 1,000 central-line days), well above that reported by the National Nosocomial Infection Surveillance (NNIS) system (*3*,*5*,*30*). This difference is due to the proportion of clinical sepsis, 80% in our study and 8% in NNIS (*3*). When microbiologically documented BSI alone is considered, our BSI rate is comparable to that reported in the literature, including the rate reported by NNIS hospitals (*5*,*14*,*16*,*31*). Surveillance estimates the incidence of a disease. Underdetection of clinical sepsis will grossly underestimate its incidence, and data generated by the system will be misinterpreted, affecting the allocation of resources. Finally, demonstrating the effectiveness of a prevention program that aims to reduce BSI will require a much greater sample size than if cases of clinical sepsis were considered in the surveillance system. Conversely, the cost-effectiveness of prevention programs will be underestimated if only microbiologically documented BSI is considered.

This study has some limitations. Whether our results can be extrapolated to other ICUs needs to be tested. Indeed, the surveillance criteria for clinical sepsis might be sensitive to local case management policies, for instance, regarding antimicrobial drug prescription. In addition, the situation in surgical ICUs might be quite different, as systemic inflammatory reactions after surgery that mimic clinical sepsis are frequent (*22*,*23*). Neither can we rule out some degree of misclassification of clinical sepsis that is actually catheter infection. This possibility is suggested by the fact that catheter exit-site infections were more prevalent in the group of patients with clinical sepsis than in the group of patients with microbiologically documented BSI. This misclassification would be very important if we were investigating the impact of clinical sepsis. However, this is not relevant in terms of surveillance and infection control and prevention because both clinical sepsis and catheter infection have the same risk factors, are sensitive to the same prevention strategies, and are equal markers of poor quality of care.

In conclusion, clinical sepsis is an epidemiologically important syndrome. We believe that surveillance strategies that can detect this syndrome should be favored because prevention, benchmarking, program evaluation, and ultimately, quality of patient care depend on the accuracy of surveillance data.
